# Effect of the loading condition on the statistics of crackling noise accompanying the failure of porous rocks

**DOI:** 10.1098/rsos.230528

**Published:** 2023-11-22

**Authors:** Csanád Szuszik, Ian G. Main, Ferenc Kun

**Affiliations:** ^1^ Department of Theoretical Physics, Doctoral School of Physics, Faculty of Science and Technology, University of Debrecen, PO Box 400, 4002 Debrecen, Hungary; ^2^ School of Geosciences, University of Edinburgh, Edinburgh EH9 3FE, UK; ^3^ Institute for Nuclear Research (Atomki), PO Box 51, 4001 Debrecen, Hungary

**Keywords:** tensile failure, crackling noise, geomaterials, discrete element simulations

## Abstract

We test the hypothesis that loading conditions affect the statistical features of crackling noise accompanying the failure of porous rocks by performing discrete element simulations of the tensile failure of model rocks and comparing the results to those of compressive simulations of the same samples. Cylindrical samples are constructed by sedimenting randomly sized spherical particles connected by beam elements representing the cementation of granules. Under a slowly increasing external tensile load, the cohesive contacts between particles break in bursts whose size fluctuates over a broad range. Close to failure breaking avalanches are found to localize on a highly stressed region where the catastrophic avalanche is triggered and the specimen breaks apart along a spanning crack. The fracture plane has a random position and orientation falling most likely close to the centre of the specimen perpendicular to the load direction. In spite of the strongly different strengths, degrees of ‘brittleness’ and spatial structure of damage of tensile and compressive failure of model rocks, our calculations revealed that the size, energy and duration of avalanches, and the waiting time between consecutive events all obey scale-free statistics with power law exponents which agree within their error bars in the two loading cases.

## 1. Introduction

Rocks experience complex loading conditions in nature including tension, compression and shear during their geological history. Deformation is accompanied by the release of elastic energy from micro-cracking events [1,2], collapse of voids in porous rocks [3], or rearrangement of particles in sheared particle packings [4], all of which can in principle be registered in the form of acoustic waves [1,5]. Acoustic emissions (AE) are the primary source of information about the microscopic processes of fracturing [5–8] providing us with valuable data about the temporal and spatial evolution of the ensemble of micro-cracks including the ability to discriminate between micro-cracking events dominated either by local tension or shear [9]. In a recent breakthrough, the evolution of the spatial structure of damage can now be seen directly by synchrotron micro-computer tomography imaging of live tests [10,11]. The acoustic ‘crackling noise’ generated during the compressive failure of rocks in laboratory experiments has been found to exhibit scale-free statistics similar to earthquakes, consistent with the universality of cracking phenomena across a broad range of length scales [2,4,12–16]. Experiments on the compressive failure of porous rocks have provided evidence that the final collapse is preceded by an accelerated crackling activity which may allow for forecasting the imminent event of ultimate collapse under certain conditions, notably for smaller sample sizes, more rapid deformation rates and more heterogeneous materials [7,17–19]. For example, detailed experiments on geothite revealed that samples with a high a porosity showed precursory acceleration [3,8]; however, the effect was completely missing in case of low porosities [20]. Systematic studies controlling the structure of samples confirmed that the degree of structural heterogeneity plays an essential role in determining the amount of crackling events increasing the reliability of failure forecast methods for materials of higher disorder [21]. These experimental findings imply that the acoustic monitoring of crackling activity, combined with other methods, can in principle be used to predict the collapse of e.g. mines where porous rocks such as sandstone and coal are the most relevant materials [18,22]. On the other hand, catastrophic failure events during deformation at slow driving rate produces more sudden onset failure [23], consistent with the absence of systematic precursors to large earthquakes [24].

Despite a large amount of experimental and theoretical effort, the effect of the loading conditions on the statistical features of crackling bursts generating AE is still a significant open question. Recently, computer simulations of discrete models of heterogeneous materials have provided a deeper understanding of the statistics and dynamics of crackling noise for different material properties, initial and boundary conditions at least in numerical tests. In particular, large-scale simulations of the failure process of lattices of electric fuses [25–27], springs [28], fibre bundles [29,30] and cohesive granular materials [31,32] all revealed a high degree of robustness of the statistics of bursts of local failures with respect to the amount of material disorder, but to date no systematic studies have been performed to isolate the effect of different loading conditions, specifically to investigate any systematic differences owing to tensile or compressive loading.

Here, we use discrete element simulations to analyse the statistical and dynamical features of crackling noise emerging during the tensile failure of a realistic model rock, and compare the results to those from simulations obtained under compressive loading of the same samples. This provides a controlled numerical test for the effect of tensile or compressive loading alone. To obtain a computer representation of sedimentary rocks, we construct cylindrical shaped rock samples by simulating the sedimentation process of particles, and connect them by beam elements in the final packing, which captures the cementation of the material. We demonstrate that under uniaxial tensile loading the model rock samples have a quasi-brittle behaviour where the fluctuating ultimate strength and the strain where cracking begins are both described by Weibull distributions. Simulations revealed that as the specimen is slowly elongated under tension, fracturing proceeds in bursts of micro-cracking events which have scale-free statistics, i.e. the size, duration and energy released by breaking avalanches are all power law distributed with a finite size cut-off. The beginning of the failure process was found to be dominated by the disordered micro-structure of the material which gives rise to random nucleation of small-sized cracks all over the sample. Approaching failure, breaking avalanches tend to localize and merge into a sharply defined fracture plane along which the specimen falls apart. With a careful numerical analysis we provide a quantitative characterization of the fluctuating sharpness, orientation, and position of the fracture plane. We then compare the results to the outcomes of the simulations of uniaxial compressive failure of the same starting specimens [31,32]. In spite of the substantial differences of the spatial structure of damage in the two cases, the statistical properties of the avalanche populations exhibit the same qualitative trends. Moreover, the values of the scaling exponents are similar to two significant figures, and indistinguishable within the error of estimation. Thus we can reject the hypothesis that loading conditions significantly affect the statistical features of crackling noise. By contrast, the bulk mechanical properties are significantly different—the ultimate strength is much lower in tension, and the bulk material yield stress is much closer to its ultimate strength, indicating a higher degree of ‘brittleness’ associated with lower predictability of failure time. Finally, the most likely orientation for the zone of localized deformation just below macroscopic failure is different from but sub-parallel to the continuum solution in tension owing to material disorder, and different from that in compression, which is controlled instead by internal friction.

## 2. Discrete element model of porous rocks

**Figure 1.**
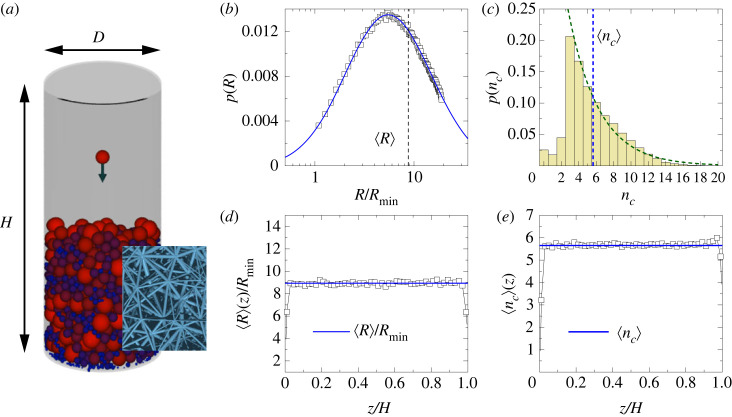
(*a*) To mimic the structure of sedimentary rocks, random homogeneous packings of particles were generated by simulating the sedimentation process inside a cylindrical container of height *H* and diameter *D*. The colour code corresponds to the particle radius *R* in such a way that dark blue and red indicate the smallest and the largest particles, respectively. The inset presents a magnified view on the beam lattice, which is constructed by a Delaunay triangulation of the final packing. The thickness of beams is scaled down to make the structure visible. (*b*) The radius *R* of particles was sampled from a lognormal distribution *p*(*R*) over the range *R*_min_ ≤ *R* ≤ *R*_max_, where *R*_max_ = 20*R*_min_ was set. (*c*) Probability distribution *p*(*n*_*c*_) of the number of contacts *n*_*c*_ of particles in the final packing. The decreasing dashed line represents an exponential which gives a reasonable description of the histogram for *n*_*c*_ ≥ 3. The average radius 〈*R*〉(*z*) (*d*) and contact number 〈*n*_*c*_〉(*z*) (*e*) of particles measured along the height *z* of the cylinder. The horizontal lines represent the corresponding sample averages 〈*R*〉 and 〈*n*_*c*_〉.

To study crackling bursts generated during the tensile failure of porous rocks, we use a discrete element model (DEM) which has been introduced recently as a generic modelling framework for heterogenous materials with a porous micro-structure [31–33]. In the model cylindrical shape, specimens are considered with diameter *D* and height *H* having the aspect ratio *H*/*D* = 2.3 typical for experimental set-ups with geo-materials [9]. The disordered micro-structure of sedimentary rocks is captured by sedimenting spherical particles in the cylinder with a random radius *R*. For this purpose, discrete element simulations were performed settling particles one-by one inside a cylindrical container under the effect of gravity. Particles lost their kinetic energy by dissipative collisions with the particles of the growing sediment layer and with the container wall until they came to rest in their final position (see figure 1*a* for illustration). We apply a soft particle contact model where particles overlap when pressed against each other giving rise to a repulsive force [34]. Particles of radii *R*_*i*_ and *R*_*j*_ and positions ***r***_*i*_ and ***r***_*j*_ overlap each other when the overlap distance *ξ* = *R*_*i*_ + *R*_*j*_ − *r*_*ij*_ has a positive value *ξ* > 0, where *r*_*ij*_ = |***r***_*i*_ − ***r***_*j*_| denotes the distance of the particles. The emerging contact force $F_{ij}^{c}$ is given by the Hertz contact law including a viscoelastic dissipation term:

2.1 $$F_{ij}^{c}=-k_{ij}^{p}(\xi^{3/2}+a\sqrt{\xi }\dot{\xi })n_{ij}.$$

The stiffness of the contact $k_{ij}^{p}$ depends on the geometry and material properties of the particles $k_{ij}^{p}=2E_{p}\sqrt{R_{ij}^{\mathrm{eff}}}/3(1-\nu_{p}^{2})$, where the effective radius $R_{ij}^{\mathrm{eff}}$ has the form $1/R_{ij}^{\mathrm{eff}}=1/R_{i}+1/R_{j}$. The parameters *E*_*p*_ and *ν*_*p*_ denote the Young modulus and Poissonian number of the material of particles, and the unit vector ***n***_*ij*_ points from particle *j* to *i*. The equation of motion of the particles was solved by a 5th order predictor-corrector scheme [35], which generated ballistic trajectories as the particles dissipated their kinetic energy over a sequence of collisions starting with a zero initial speed at a random position above the growing sediment layer. The simulation stopped when the required number of particles *N* was reached in the sediment with a smooth upper surface.

In the simulations, the radius of particles *R* was sampled from a lognormal distribution commonly found for granules in sedimentary rocks. The range of particle radius was bounded *R*_min_ ≤ *R* ≤ *R*_max_ since very small particles may settle to the bottom of the container bouncing through the void space between the large ones. To avoid this size segregation, the ratio of the largest *R*_max_ and smallest *R*_min_ radii was set to *R*_max_/*R*_min_ = 20, while the average particle radius 〈*R*〉 was fixed to the value 〈*R*〉 = 8.9*R*_min_ for all the samples [31]. Figure 1*b* demonstrates the size distribution *p*(*R*) of particles in the final packing, which perfectly agrees with the prescribed lognormal form. To characterize the internal structure of the sample, we determined the probability distribution *p*(*n*_*c*_) of the number of contacts *n*_*c*_ of the particles. The probability distribution (figure 1*c*) for *n*_*c*_ can be described by an exponential

2.2 $$p(n_{c})∼e^{-n_{c}/\left\langle n_{c}\right\rangle },$$

with an average contact number 〈*n*_*c*_〉 ≈ 5.6, in a reasonable agreement with measurements on sedimentary rocks [36]. Particles with a few contacts *n*_*c*_ = 0, 1, 2 are typically small ones either lying at the bottom of the container or along the vertical wall. To test the homogeneity of the particle packing, we determined the average radius 〈*R*〉(*z*) and average contact number 〈*n*_*c*_〉(*z*) of particles as a function of height *z* measured along the cylinder axis from the bottom circle. Figure 1*d*,*e* shows that both quantities 〈*R*〉(*z*) and 〈*n*_*c*_〉(*z*) fluctuate close to their sample average which implies a high degree of homogeneity. In our sedimentation technique, the value of the porosity, i.e. the average fraction of voids of the sample can be controlled by varying the width of the distribution *p*(*R*) of the particle radius [37]. With the set-up used in the present study the samples’ porosity had mild fluctuations around the average 0.41.

To represent the cementation between granules, a Delaunay triangulation was performed with the centre of spheres in the final packing, and the particle centres were connected by beam elements along the edges of the triangles. The geometrical features of beams are determined by the random particle packing in such a way that the equilibrium length $l_{ij}^{0}$ of the beam between particles *i* and *j* is the distance of the particle centres in the initial configuration $l_{ij}^{0}=|r_{i}^{0}-r_{j}^{0}|$, while the beam cross section *S*_*ij*_ is calculated as $1/S_{ij}=1/(R_{i}^{2}\pi )+1/(R_{j}^{2}\pi )$. It follows that the heterogeneous micro-structure of the particle packing gives rise to randomness of the beam geometry which in turn affects the values of the physical quantities, e.g. stiffness of beams, as well. A magnified view of a small part of the beam lattice attached to the particles is highlighted by the inset of figure 1*a*. In the model, we implemented a beam dynamics based on Euler–Bernoulli beams as described in [34,38,39]. A quantitative estimate of the deformation of beams is obtained from a local coordinate system attached to both particles at the beam ends. As the particles undergo translational and rotational motion during the deformation of the sample, the beams suffer elongation, compression, shear and torsion, resulting in forces and torques on the particles. The axial force $F_{ij}^{b}$ exerted on particle *i* by the beam connecting particles *i* and *j* is controlled by the beam elongation $\Delta l_{ij}=r_{ij}-l_{ij}^{0}$ in the form

2.3 $$F_{ij}^{b}=-k_{ij}^{b}\Delta l_{ij}n_{ij}.$$

The stiffness of beams $k_{ij}^{b}$ is determined by the Young modulus *E*_*b*_ and the geometrical features of the beams represented by the term $k_{ij}^{b}=E_{b}S_{ij}/l_{ij}^{0}$. A dissipative component of the force is also added to equation (2.3) similar to that used in the sedimentation simulations, equation (2.1). The flexural forces and torques can be determined by keeping trace of the change of the orientation of beam ends with respect to the body fixed coordinate system $e_{x}^{b}$, $e_{y}^{b}$, $e_{z}^{b}$ of the particles, where $e_{x}^{b}$ is aligned with the beam orientation [34]. In a simple case when both beam ends rotate around the $e_{z}^{b}$ axis of the body fixed system by angles $\Theta_{i}^{z}$ and $\Theta_{j}^{z}$, the resulting force $Q_{i}^{z,b}$ and torque $M_{i}^{z,b}$ acting on particle *i* can be cast into the form [34]

2.4 $$Q_{i}^{z,b}=3E_{b}I_{ij}\frac{\Theta_{i}^{z}+\Theta_{j}^{z}}{{\left(l_{ij}^{0}\right)}^{2}}e_{y}^{b}$$

and

2.5 $$M_{i}^{z,b}=E_{b}I_{ij}\frac{\Theta_{i}^{z}+\Theta_{j}^{z}}{l_{ij}^{0}}e_{z}^{b}+(Q_{i}^{z,b}\times |r_{ij}|e_{x}^{b}),$$

where *I*_*ij*_ denotes the beam’s moment of inertia. Torsion arises owing to the relative rotation around the $e_{x}^{b}$ axis which gives rise to the moment

2.6 $$M_{i}^{x,b}=G_{b}I_{ij}^{t}\frac{\Theta_{i}^{x}-\Theta_{j}^{x}}{l_{ij}^{0}}e_{x}^{b}.$$

Here, *G*_*b*_ denotes the shear modulus of the beam, and $I_{ij}^{t}$ is the torsional moment of inertia with respect to the beam axis. Beam forces and torques were transformed to the global coordinate system of the particle packing where the equation of motion is solved numerically for the translational and rotation degrees of freedom [35]. The same fifth-order predictor-corrector solver is used for the simulations as for the generation of the initial particle packing taking into account the boundary and loading conditions [35].

As the specimen deforms under an externally exposed loading, beams break when their local strength is equalled or exceeded, leading to the formation of micro-cracks. We use a physical breaking criterion which allows beam breaking either by stretching or bending:

2.7 $${\left(\frac{\varepsilon_{ij}}{\varepsilon_{th}}\right)}^{2}+\frac{\max(|\Theta_{i}|,|\Theta_{j}|)}{\Theta_{th}}\geq 1,$$

where $\varepsilon_{ij}=\Delta l_{ij}/l_{ij}^{0}$ is the axial strain of the beam between particles *i* and *j*, while $\Theta_{i}$ and $\Theta_{j}$ denote the generalized bending angles at the two beam ends. The breaking parameters $\varepsilon_{th}$ and $\Theta_{th}$, which control the relative importance of the two breaking modes, have fixed values for all the beams $\varepsilon_{th}=0.003$ and $\Theta_{th}=2^{\circ }$, however, the structural randomness of the particle packing generates emergent disorder, e.g. in the stiffness parameters of beam elements. Those particles which are not coupled by beams interact through contact forces where the normal force is modelled by the law given by equation (2.1), while for the tangential force the Coulomb friction law is applied with a friction coefficient of *μ* = 0.5 [31–34].

**Figure 2.**
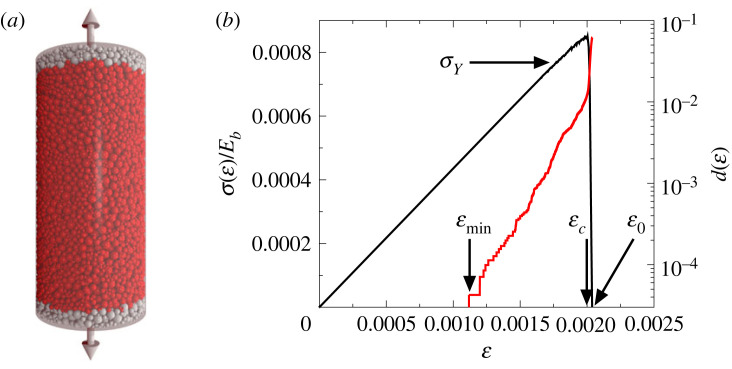
(*a*) Set-up of the numerical experiments. DEM simulations were performed by slowly elongating cylindrical samples composed of a random packing of spherical particles. A few particle layers (highlighted in grey) were clamped at the top and bottom of the sample which were then slowly moved against each other along the cylinder axis. (*b*) Constitutive curve σ(ε) (black) of the system together with the accumulated damage d(ε) (red) in a single sample. The stress *σ* is scaled with the Young modulus of beams *E*_*b*_. The arrows indicate the strain values where cracking starts $\varepsilon_{\min}$, where final breakdown occurs $\varepsilon_{c}$, and where the stress drops to zero after failure $\varepsilon_{0}$, furthermore, the yield stress *σ*_*Y*_, where the first discernible deviation from linearity of the σ(ε) curve occurs.

Tensile loading of the cylindrical samples with deformation control was performed by clamping a few boundary layers of particles at the bottom and top of the cylinder, which were then slowly moved further apart along the cylinder axis at a constant speed *v*_0_, resulting in a constant strain rate $\dot{\varepsilon }$. On the side wall of the cylinder no confining pressure was applied. The breaking criterion, equation (2.7) was evaluated in each iteration step and those beams which fulfil the condition were removed from the sample. As a result of consecutive beam breakings, cracks are formed and the sample eventually breaks apart, when these grow, coalesce and localize on a sub-planar deformation zone containing the surface of the eventual macroscopic fracture. The simulation stops when the force acting on the clamped layer of particles drops down to zero which marks the point where the specimen disintegrates. The initial sample and the loading condition are illustrated in figure 2*a*. The model has been successfully used to study the failure process of porous rocks under compressive loading where it closely reproduced the experimental findings on the spatial structure of damage and on the intermittent breaking activity accompanying the failure process and the relevant scaling exponents for the crackling noise [31–33,37]. Here, we repeat these simulations in exactly the same way, changing only the external loading condition from compression to tension.

To match our previous simulations in compression, we considered cylindrical samples comprising on average *N* ≈ 20.000 particles but this time under tensile load. We then compare the results with those from previously published simulations on uniaxial compressive loading. Unless stated otherwise, the results presented here refer to new results obtained from the tensile load case. The diameter *D* of the base circle of the sample is *D* ≈ 87〈*R*〉, where 〈*R*〉 denotes the average radius of the spherical particles. We generated a good statistical sample of the behaviour from over *K* = 1000 simulations using different starting samples created by independent simulations of the sedimentation process. The parameter values of the simulations and the notation of characteristic quantities of the system are summarized in table 1. For further details of the modelling approach, see [31,33,37].

**Table 1.** Summary of the notation of characteristic quantities of the system, and the parameter values of DEM simulations.

parameter	notation	value	unit
beams			
*E*_*b*_	Young modulus	6	GPa
*G*_*b*_	shear modulus	6	GPa
$\varepsilon_{th}$	breaking threshold	0.003	—
$\Theta_{th}$	breaking threshold	2	°
particles			
〈*R*〉	average radius	0.1	mm
*ρ*	density	3000	kg m^−3^
*E*_*p*_	particle Young modulus	6	GPa
*ν*_*p*_	particle Poission ratio	0.3	—
*μ*	Coulomb friction coefficient	0.5	—
loading			
Δ*t*	time step	10^−8^	s
$\dot{\varepsilon }$	strain rate	0.01	1 s^−1^
notation for macroscopic response			
*σ*	stress		
ε	strain		
*Y*_eff_	effective Young modulus		
*σ*_*c*_, $\varepsilon_{c}$	failure stress, strain		
*σ*_*Y*_	yield stress		
$\sigma_{\min},\varepsilon_{\min}$	stress, strain at first micro-crack		
$\varepsilon_{0}$	post-peak strain where *σ* falls to zero		

## 3. Quasi-brittle response

We performed computer simulations of the uniaxial tensile loading of cylindrical specimens to monitor their macroscopic response and the underlying microscopic process of fracturing. To characterize the mechanical response of the sample under deformation controlled loading, we measured the force *F* needed to maintain the deformation as the clamped boundary particle layers were slowly moved along the cylinder axis. The macroscopic stress *σ* and strain ε of the sample were obtained as *σ* = *F*/*A* and ε=ΔH/H, where *A* = *πD*^2^/4 is the initial cross-section of the cylinder, and Δ*H* is the elongation of the sample. As the sample is slowly elongated individual beams, representing local cohesive particle contacts, gradually break and form cracks which eventually leads to global failure when the specimen breaks apart into two large pieces. The simulation stops when the force *F* acting on the boundary layers drops down to zero. Figure 2*b* presents the constitutive curve σ(ε) for a single sample, i.e. the stress–strain curve following the evolution of the system up to final breakdown. The system has a linearly elastic behaviour over a broad range of ε and nonlinearity is only observed in the vicinity of the maximum stress *σ* after which *σ* falls to zero. The overall strength of the sample can be characterized by the position $\varepsilon_{c}$ and value *σ*_*c*_ of the maximum of the σ(ε) curve, which define the critical strain and the critical stress of the system, respectively.

**Figure 3.**
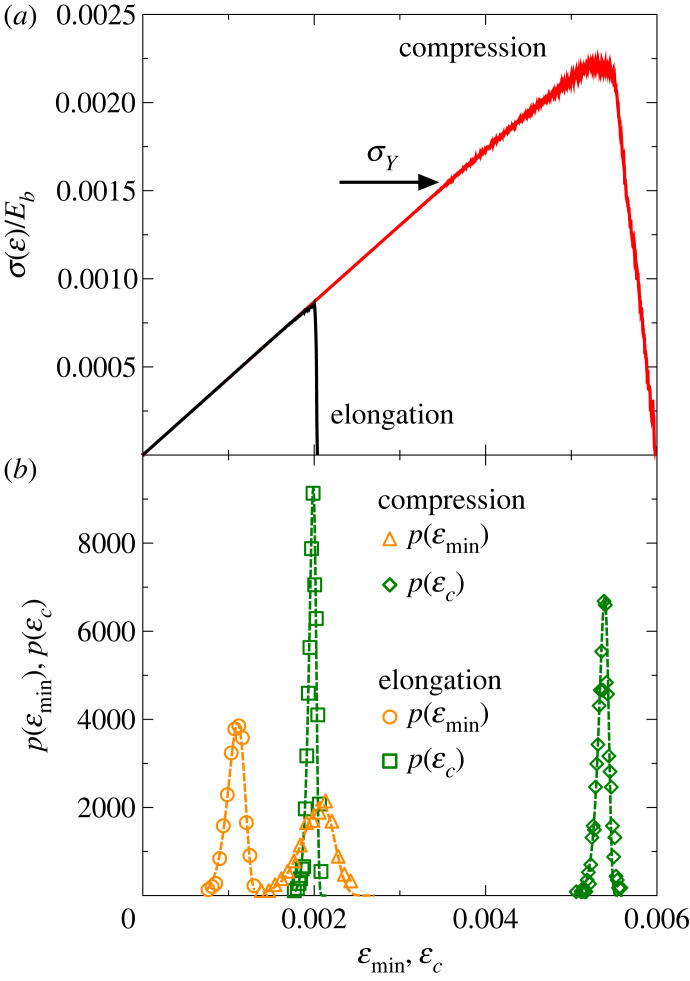
(*a*) Stress–strain evolution σ(ε) of a representative sample measured under uniaxial compression (red) and elongation (black). The stress *σ* is scaled with the Young modulus of the beams *E*_*b*_. The horizontal arrow indicates the yield stress *σ*_*Y*_ of the compressive case. (*b*) The incremental probability distributions $p(\varepsilon_{\min})$ and $p(\varepsilon_{c})$ of the strain of crack initiation $\varepsilon_{\min}$ and of global failure $\varepsilon_{c}$ for both loading cases. The dashed lines represent best fits of the results assuming a Weibull distribution, equation (3.1). To facilitate the comparison of strain values, the horizontal axis of (*a*) and (*b*) are the same.

The state of damage can be quantified by the fraction of broken contacts *d* = *N*_*br*_/*N*_*B*_, where *N*_*br*_ and *N*_*B*_ denote the number of broken beams and the total number of intact ones in the initial state, respectively. Comparing the damage curve d(ε) to the evolution of the mechanical response σ(ε), figure 2*b* shows that the growing nonlinearity of σ(ε) is caused by the acceleration of the cumulative damage as the critical point is approached. The relatively weak nonlinearity and the sudden stress drop at global failure imply a quasi-brittle response of the model rock. The tensile and compressive responses of the same specimen are compared in figure 3*a*, where all the model parameters had the same values in the simulations, but where the direction of the motion of the boundary particle layers were opposite to each other. The two σ(ε) curves fall on the top of each other, which confirms that the effective Young modulus *Y*_eff_, i.e. the slope of the linear regime of σ(ε), does not depend on the loading condition. Careful fitting of the constitutive curves yields *Y*_eff_/*E*_*b*_ ≈ 0.22. However, the ultimate strength defined by the position of the maximum stress at $\varepsilon_{c}$ and *σ*_*c*_, where global failure occurs proved to be significantly higher under compression owing to the stabilizing effect of closing cracks, primarily perpendicular to the direction of maximum principal stress. This greater strength in compression than tension has been known empirically since Roman times, when the arch replaced the much weaker pillar and beam design of earlier Greek architecture. The stress drop at global failure is less abrupt for compression and failure is preceded by a stronger nonlinearity than for tensile loading implying a higher degree of ‘brittleness’ (defined below) of the sample in the tensile case.

The disordered micro-structure of the sample controls the strain of the onset of local cracking $\varepsilon_{\min}$ and the macroscopic yield stress *σ*_*Y*_, furthermore, the critical strain $\varepsilon_{c}$ and stress *σ*_*c*_ of ultimate failure are stochastic quantities with sample-to-sample fluctuations. To give a measure of these fluctuations, we determined the probability distributions $p(\varepsilon_{\min})$ and $p(\varepsilon_{c})$ for both tensile and compressive loading, which are presented in figure 3*b*. The relative variances have the values $var(\varepsilon_{\min})/\left\langle \varepsilon_{\min}\right\rangle \approx 0.09$ and $var(\varepsilon_{c})/\left\langle \varepsilon_{c}\right\rangle \approx 0.025$ (tension), $var(\varepsilon_{\min})/\left\langle \varepsilon_{\min}\right\rangle \approx 0.105$ and $var(\varepsilon_{c})/\left\langle \varepsilon_{c}\right\rangle \approx 0.014$ (compression), which indicate that both quantities $\varepsilon_{\min}$ and $\varepsilon_{c}$ have mild fluctuations at the system size *N* considered. Numerical analysis revealed that for both loading cases the probability distributions $p(\varepsilon_{\min})$ and $p(\varepsilon_{c})$ can be well fitted by the Weibull distribution:

3.1 $$p(x)=\frac{m}{\lambda }{\left(\frac{x}{\lambda }\right)}^{m-1}e^{-{\left(x/\lambda \right)}^{m}},$$

with two parameters, where *λ* sets the scale of the values of *x*, while the exponent *m* controls the shape of the distribution. Best fits presented in figure 3*b* were obtained with equation (3.1) using the parameter values *λ* = 0.0012, *m* = 11 for $\varepsilon_{\min}$, and *λ* = 0.00204, *m* = 50 for $\varepsilon_{c}$, and *λ* = 0.00209, *m* = 12 for $\varepsilon_{\min}$, and *λ* = 0.0054, *m* = 99 for $\varepsilon_{c}$, for tensile and compressive loading, respectively. The critical load *σ*_*c*_ was found to obey the same statistics with similarly high Weibull exponents both for tension and compression. The high Weibull exponents are consistent with the mild fluctuations in the emergent parameters between starting samples quantified by the relative variances above. The modal values of the strain of crack initiation $\varepsilon_{\min}$ and ultimate failure $\varepsilon_{c}$ are 0.00112, 0.00199, and 0.00219, 0.00536 for tensile and compressive loading, respectively. It follows from the numerical analysis that the average strength of the sample is about 2.7 times higher under compression than under tensile loading, although the relative fluctuations of the strength values are nearly the same, in agreement with experimental findings [40].

**Figure 4.**
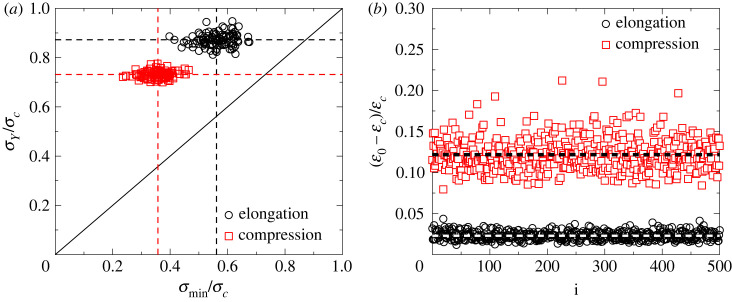
Pre-peak (*a*) and post-peak (*b*) quantities characterizing the degree of brittleness of the sample. Scatter plots of the yield stress *σ*_*Y*_ versus the stress of crack initiation *σ*_min_ (*a*), and of the difference of the strain of ultimate failure $\varepsilon_{c}$ and of the state where the stress falls to zero after failure $\varepsilon_{0}$ (*b*) are presented for tensile and compressive loading. In (*a*), the dashed lines indicate the average values 〈*σ*_*Y*_/*σ*_*c*_〉 and 〈*σ*_min_/*σ*_*c*_〉, while the continuous line represents the *y* = *x* function to demonstrate that the relation *σ*_*Y*_ > *σ*_min_ always holds. In (*b*), the dashed horizontal lines indicate the average values $\left\langle (\varepsilon_{0}-\varepsilon_{c})/\varepsilon_{c}\right\rangle$ for tension and compression.

To characterize the degree of brittleness of the sample, we used two quantities: the pre-peak brittleness of the macroscopic response is quantified by the ratio of the yield stress *σ*_*Y*_ and of the critical stress *σ*_*c*_ of ultimate failure, where *σ*_*Y*_ is obtained as the stress where the first discernible deviation of the σ(ε) curve occurs from the linear behaviour. We also measured the difference of the critical strain $\varepsilon_{c}$ and the strain $\varepsilon_{0}$ where the stress drops to zero after failure. (For a definition of $\varepsilon_{0}$, see figure 2*b*.) The ratio $(\varepsilon_{0}-\varepsilon_{c})/\varepsilon_{c}$ characterizes how fast the σ(ε) curve falls towards zero so that it serves as a post-peak brittleness parameter. Figure 4*a* shows *σ*_*Y*_/*σ*_*c*_ as a function of *σ*_min_/*σ*_*c*_ in the form of a scatter plot where each symbol represents a single sample. In figure 4*b*, a similar scatter plot of the post-peak brittleness parameter is presented as a function of the order number *i* of the samples. All quantities have relatively low sample-to-sample variations fluctuating around well-defined averages. Comparing the average ratios 〈*σ*_*Y*_/*σ*_*c*_〉 obtained for tension 〈*σ*_*Y*_/*σ*_*c*_〉 ≈ 0.89 and for compression 〈*σ*_*Y*_/*σ*_*c*_〉 ≈ 0.72, there is a significantly higher degree of pre-peak brittleness of the samples under tension associated with a higher ratio 〈*σ*_*Y*_/*σ*_*c*_〉. This is accompanied by a higher degree of post-peak brittleness, i.e. in tension the stress significantly faster goes to zero after failure with $\left\langle (\varepsilon_{0}-\varepsilon_{c})/\varepsilon_{c}\right\rangle \approx 0.02$ than for compression where $\left\langle (\varepsilon_{0}-\varepsilon_{c})/\varepsilon_{c}\right\rangle \approx 0.12$ was obtained.

## 4. Scale-free bursting activity

Simulations revealed that damaging of the sample proceeds in intermittent bursts of beam breakings, which can be considered as the counterparts of AE sources in real experiments. The reason is that the breaking of a beam is followed by the redistribution of stress in the sample, which can relax but also in turn increase the load on beams in certain regions of the local neighbourhood thereby triggering additional failure events, which are again followed by load redistribution. Thus a single beam breaking may trigger an entire avalanche of breaking events which either stops when all the beams can sustain the local load inside the sample or leads to catastrophic failure of the system. The time scale *t*_*c*_ of load redistribution is controlled by the speed of elastic waves of the sample. In order to identify avalanches of local breaking events, we follow the techniques developed in [31,32]: in the simulations, we record the time *t*_*j*_ of all individual beam breakings and assume that those breaking events which follow each other within the correlation time *t*_*j*+1_ − *t*_*j*_ < *t*_*c*_ belong to the same trail of breakings. Single avalanches determined by the algorithm are characterized by their size Δ, duration *T*, spatial position ***r*** and energy *E* dissipated during the avalanche. The burst size Δ is obtained as the total number of beams breaking in the bursts, which is proportional to the newly created free surface inside the sample. The spatial position ***r*** of an avalanche of size Δ is calculated as the centre of mass position of the cloud of beams:

4.1 $$r=\frac{\sum_{\;j=1}^{\Delta }r_{j}^{b}}{\Delta },$$

where $r_{j}^{b}$ (*j* = 1, …, Δ) denotes the position vector of the centre of beams breaking in the avalanche. The burst duration *T* is calculated as the time difference between the last and the first breakings of the avalanche:

4.2 $$T=t_{\mathrm{last}}-t_{\mathrm{first}},$$

while the dissipated energy *E* is obtained as the sum of the elastic energies stored in the deformation of beams at the time of their breaking.

**Figure 5.**
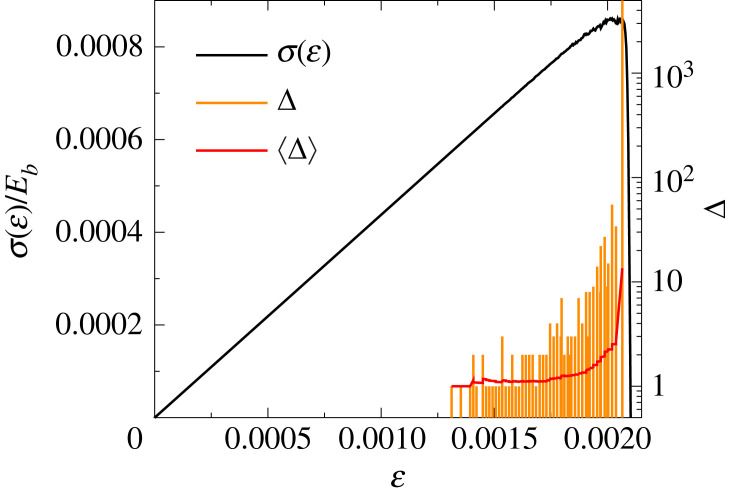
Sequence of breaking avalanches of beams along with the constitutive curve σ(ε) of a representative sample. The height of the bars represents the size Δ_*i*_ of the avalanches *i* = 1, …, *n*_*b*_. The continuous red line indicates the moving average of the avalanche size 〈Δ〉 obtained by averaging over 51 consecutive events.

**Figure 6.**
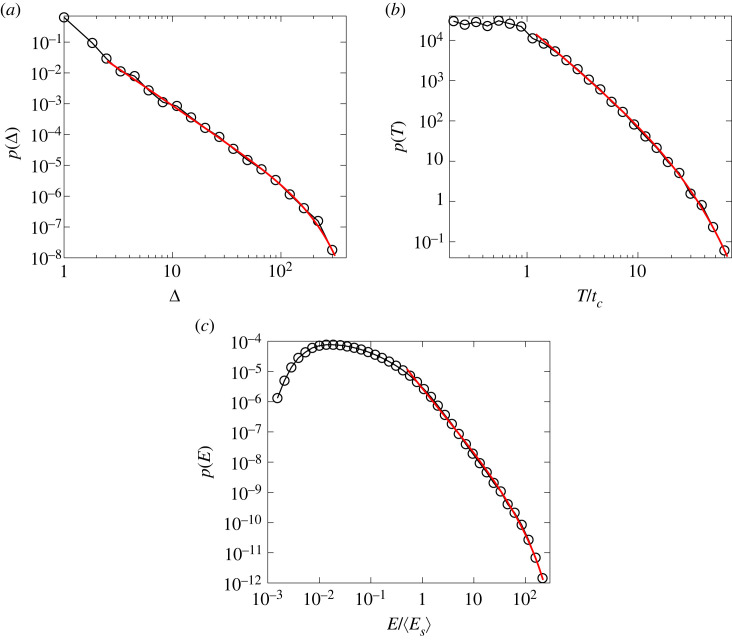
Probability distribution of the size *p*(Δ) (*a*), duration *p*(*T*) (*b*) and dissipated energy *p*(*E*) (*c*) of bursts taking into account all the events which occurred before catastrophic collapse (up to the maximum of the constitutive curve σ(ε)). The continuous red lines represent fits with the functional form equation (4.3). The duration *T* and the energy *E* are made dimensionless by division with the correlation time *t*_*c*_ and the average energy 〈*E*_*s*_〉 dissipated by a single beam breaking, respectively.

The sequence of bursts is illustrated for a failure process in figure 5, where the size of bursts Δ_*i*_ (*i* = 1, …, *n*_*b*_), represented by the height of the orange bars, is plotted at the strain where the bursts occurred. At the beginning of fracturing bursts comprise only a few broken beams, however, as global failure is approached the bursts have an increasing size Δ apart from fluctuations, and they follow each other after smaller and smaller strain increments indicating the acceleration of the failure process. Owing to the inherent disorder of the structure of the sample the fluctuating burst size Δ covers a broad range, where the largest burst is always the final one, i.e. the catastrophic avalanche during which a macro-crack is formed spanning the entire sample. To quantify the statistics of the fluctuating quantities, we determined the probability distributions of the size *p*(Δ), duration *p*(*T*) and energy *p*(*E*) of burst considering all events except for the catastrophic one. The double logarithmic plots of figure 6 show that for sufficiently large values of Δ, *T* and *E* the three distributions are well approximated by a power law functional form followed by an exponential cutoff owing to the finite sample size

4.3 $$p(x)∼x^{-\tau }\exp\left(-x/x_{0}\right).$$

Here, *x*_0_ denotes the characteristic value of the avalanche quantity controlling the cut-off. The value of the power law exponent *τ* was obtained by fitting the curves in figure 6*a*–*c* as *τ*_Δ_ = 2.4 ± 0.11, *τ*_*T*_ = 2.25 ± 0.08 and *τ*_*E*_ = 2.11 ± 0.05 for the distributions of the burst size *p*(Δ), duration *p*(*T*), and dissipated energy *p*(*E*), respectively. In the case of the dissipated energy, the local maximum of *p*(*E*) is caused by the energy distribution of single-breaking beams.

**Figure 7.**
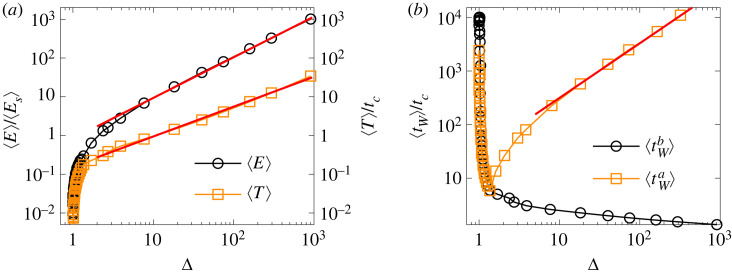
(*a*) Average energy 〈*E*〉 and duration 〈*T*〉 of avalanches of size Δ. The straight lines represent power laws of exponent *ν*_*E*_ = 1.01 and *ν*_*T*_ = 0.77. (*b*) Average value of the waiting time elapsed before $\left\langle t_{W}^{b}\right\rangle$ and after $\left\langle t_{W}^{a}\right\rangle$ avalanches of size Δ. Power law correlation is revealed between $\left\langle t_{W}^{a}\right\rangle$ and Δ with the correlation exponent *ν*_*W*_ = 1.02.

Of course, the exponents *τ*_Δ_, *τ*_*T*_ and *τ*_*E*_ are not independent of each other, avalanche quantities are usually positively correlated because avalanches of larger size typically have a longer duration and dissipate a higher amount of energy. To give a quantitative characterization of this correlation, we calculated the average duration 〈*T*〉 and energy 〈*E*〉 of bursts of a given size Δ. Figure 7*a* demonstrates that for sufficiently large burst sizes the correlation of the three quantities can be well described by power laws

4.4 $$\begin{array}{rl} & \left\langle T\right\rangle ∼\Delta^{\nu_{T}}\\ \mathrm{and}\; & \left\langle E\right\rangle ∼\Delta^{\nu_{E}}, \end{array}\}$$

where best fit of the curves was obtained with the exponents *ν*_*T*_ = 0.770 ± 0.025 and *ν*_*E*_ = 1.01 ± 0.02. The best-fit exponents *ν*_*T*_, *ν*_*E*_, *τ*_Δ_, *τ*_*T*_ and *τ*_*E*_ quoted above are consistent within error with the following equations:

4.5 $$\begin{array}{rl} & \tau_{T}=\frac{\left(\tau_{\Delta }+\nu_{T}-1\right)}{\nu_{T}}\\ \mathrm{and}\; & \tau_{E}=\frac{\left(\tau_{\Delta }+\nu_{E}-1\right)}{\nu_{E}}. \end{array}\}$$

**Figure 8.**
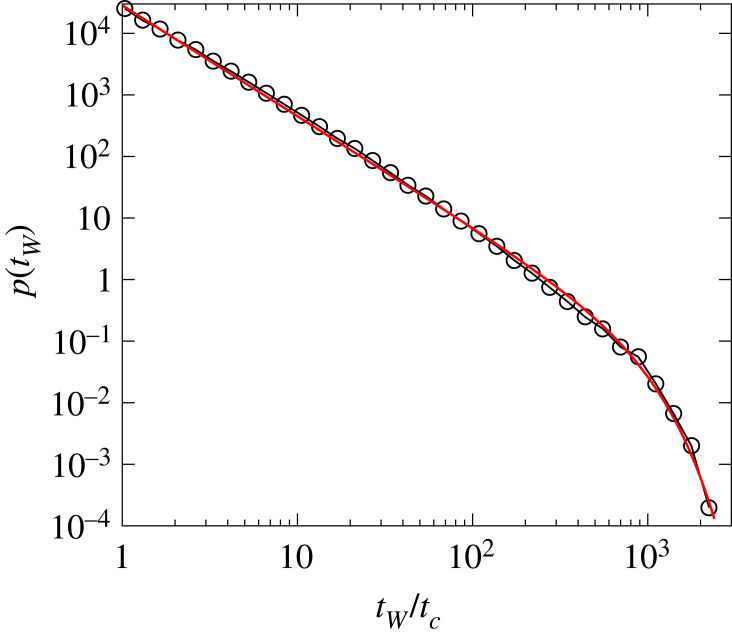
Probability distribution of waiting times *p*(*t*_*W*_) between consecutive avalanches. The red line represents the best fit obtained with equation (4.3).

For the time evolution of the intermittent avalanche sequence, it is crucial to study the statistics of waiting times *t*_*W*_, i.e. the duration of silent periods between consecutive bursts. Figure 8 demonstrates that the probability distribution of waiting times *p*(*t*_*W*_) also has a scale-free behaviour that is well described by the functional form of equation (4.3). The exponent *τ*_*W*_ of the waiting time distribution *p*(*t*_*W*_) has a relatively high value *τ*_*W*_ = 1.81 ± 0.04 which implies that large waiting times are relatively rare in the sequence. Because avalanches are driven by the gradual redistribution of load over the intact beams in the solid, a correlation may arise between the size of avalanches and the waiting time until the next avalanche is triggered. Avalanches release stress in their close vicinity so that it can be expected that after a larger avalanche one has to wait longer to build up again the stress field and initiate the next avalanche under the slowly increasing deformation controlled loading. To quantify this effect, we determined the average values of the waiting time that elapsed between consecutive events before $\left\langle t_{W}^{b}\right\rangle$ and after $\left\langle t_{W}^{a}\right\rangle$ avalanches of a given size Δ. Figure 7*b* shows that $\left\langle t_{W}^{b}\right\rangle$ rapidly converges to the vicinity of *t*_*c*_, which implies no correlation between $t_{W}^{b}$ and the size of the following burst Δ. However, $\left\langle t_{W}^{a}\right\rangle$ exhibits a power law increase with Δ:

4.6 $$\left\langle t_{W}^{a}\right\rangle ∼\Delta^{\nu_{W}},$$

which shows that it takes longer to initiate the next avalanche after a larger one. The exponent *ν*_*W*_ proved to have the value *ν*_*W*_ = 1.02 ± 0.02.

Computer simulations of uniaxial compression of the same specimens performed in [31,32] revealed the same type of scale-free statistics of avalanche characteristics. Table 2 summarizes the value of the exponents of the probability distribution of the size, energy and duration of cracking avalanches, the correlation exponents of the three quantities and the waiting time exponents for both tension and compression simulations. Owing to the higher strength and stability of the sample observed under compression, the system can tolerate a larger number of bursts *n*_*b*_ which can grow to larger sizes without becoming catastrophic. So a material is not only stronger in compression, but can also absorb more damage. Our analysis revealed that the cut-offs of the distributions of avalanche quantities, i.e. the average of the largest avalanche size 〈Δ_max_〉, energy 〈*E*_max_〉 and duration 〈*T*_max_〉, and the average number of avalanches 〈*n*_*b*_〉 are five to eight times larger under compression than under tension. From table 2, the corresponding exponents of tension and compression simulations agree with each other within the error bars except for the correlation exponents *ν*_*E*_ and *ν*_*W*_ which are smaller for tension. Despite this caveat, there is no significant difference in the scaling properties of avalanches produced in the same starting material under tension and compression.

**Table 2.** Power law exponents characterizing the statistics of breaking avalanches obtained under strain-controlled uniaxial tensile and compressive loading. Results of compression simulations are taken from [31,32].

exponent	notation	tension	compression
avalanche size	*τ*_Δ_	2.40 ± 0.11	2.22 ± 0.12
avalanche duration	*τ*_*T*_	2.25 ± 0.08	2.4 ± 0.13
avalanche energy	*τ*_*E*_	2.11 ± 0.05	2.02 ± 0.06
waiting time	*τ*_*W*_	1.81 ± 0.04	2.0 ± 0.06
size-duration	*ν*_*T*_	0.770 ± 0.025	0.8 ± 0.02
size-energy	*ν*_*E*_	1.01 ± 0.02	1.15 ± 0.03
size-waiting time	*ν*_*W*_	1.02 ± 0.02	1.37 ± 0.05

## 5. Spatial structure of damage

**Figure 9.**
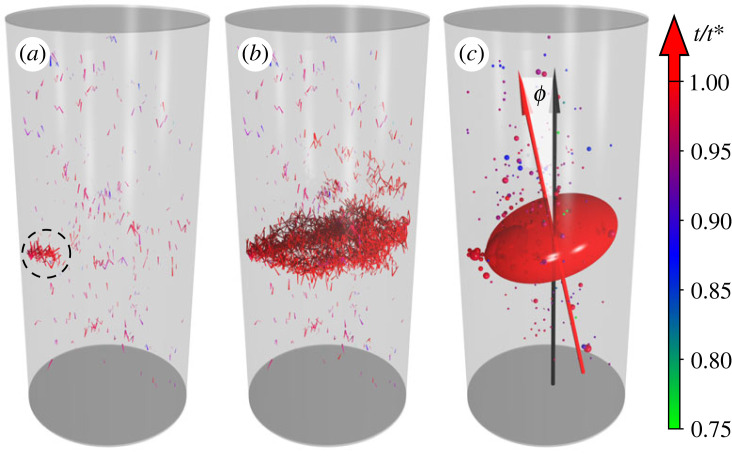
Spatial structure of damage in the specimen. (*a*) Broken beams of avalanches which occurred before the catastrophic event. The circle highlights the region where the last five avalanches occurred before the catastrophic one, this is also the region where the catastrophic avalanche was initiated. (*b*) All the broken beams including also the ones of the catastrophic avalanche. (*c*) The oblate ellipsoid obtained for the point cloud of the centre of the broken beams of the catastrophic event. Avalanches occurred before the catastrophic one are indicated by spheres with a radius proportional to the avalanche size. The colour of the spheres indicates the time *t* when the avalanche occurred according to the colour scale on the right, where *t* is scaled with the critical time t* of failure. Angle *ϕ* enclosed by the shortest axis of the ellipsoid with the load direction is also highlighted.

**Figure 10.**
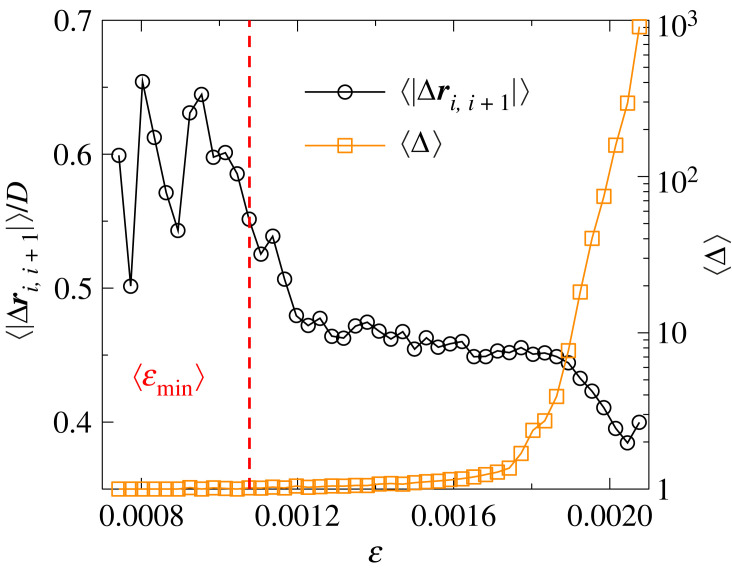
Average distance 〈|Δ***r***_*i*,*i*+1_|〉 of consecutive avalanches together with the average avalanche size 〈Δ〉 as function of ε. The vertical dashed line indicates the average value of the strain $\left\langle \varepsilon_{\min}\right\rangle$, where micro-cracking starts.

In spite of the robustness of the statistics of crackling noise, computer simulations revealed substantial differences between the spatial structure of the damage of tensile and compressive failure. As the specimen is slowly elongated first the weakest beams break, which results in random crack nucleation scattered all over the sample in an uncorrelated manner as illustrated by figure 9*a*. The early cracks have a small size, comprising only a few broken bonds, however, as fracturing proceeds the size of avalanches and the resulting cracks increase in size and their spatial appearance becomes increasingly correlated. In figure 9*a*, the spatial region where micro-cracks of the last five avalanches occurred before the catastrophic one is highlighted by a circle. A localization of damage can be inferred inside the circle which contains the nucleation point for the catastrophic avalanche. In order to characterize the spatial properties of the sequence of avalanches, we determined the average distance 〈|Δ***r***_*i*,*i*+1_|〉 of consecutive events, where Δ***r***_*i*,*i*+1_ = ***r***_*i*+1_ − ***r***_*i*_ is the relative position of two bursts with positions ***r***_*i*_ and ***r***_*i*+1_ following each other in the event sequence. In figure 10, the average distance 〈|Δ***r***_*i*,*i*+1_|〉 is re-scaled with the diameter *D* of the cylindrical sample so that the value 〈|Δ***r***_*i*,*i*+1_|〉/*D* ≈ 0.45 of the ratio indicates the random dispersion of consecutive events over the entire sample. This behaviour is characteristic for the beginning of the fracture process until avalanches have a relatively low average size 〈Δ〉. However, when 〈Δ〉 starts to increase in the vicinity of global failure, the distance of consecutive avalanches gets gradually reduced indicating the emergence of spatial correlations in agreement with the spatial clustering of damage in figure 9*a*,*b*. Note that the high values of 〈|Δ***r***_*i*,*i*+1_|〉/*D* observed below the average crack initiation strain $\left\langle \varepsilon_{\min}\right\rangle$ are caused by large sample to sample fluctuations.

Computer simulations revealed that failure of the sample occurs when avalanches become spontaneously localized in space and the broken beams form a fracture plane which spans the sample nearly perpendicular to the load direction (see figure 9*b* for illustration). The macroscopic fracture is formed within a cloud or localized damage zone of predominantly tensile micro-cracks (broken beams) which accumulate during the localized avalanches. To characterize the spatial extension and shape of this cloud, and hence, the sharpness of the fracture plane, we determined the moment of inertia matrix *I* of the set of the middle points of the broken beams. The three eigenvalues of *I*, i.e. the principal moments of inertia *A*, *B* and *C* give a measure of the extensions and shape of the point cloud. Calculations revealed that two eigenvalues have nearly the same magnitude *A* ≈ *B*, while the third one is significantly lower *C* < *A*. These relative magnitudes imply that the shape of the point cloud can be approximated by an oblate ellipsoid. As an example, figure 9*c* presents the ellipsoid obtained by this analysis where the three axes are directed along the eigenvectors of *I* and the lengths of the axes are proportional to the corresponding eigenvalues *C* < *B* < *A*. The centre of the ellipsoid is positioned to the location of the centre of mass of the point cloud of broken beams of the catastrophic avalanche.

**Figure 11.**
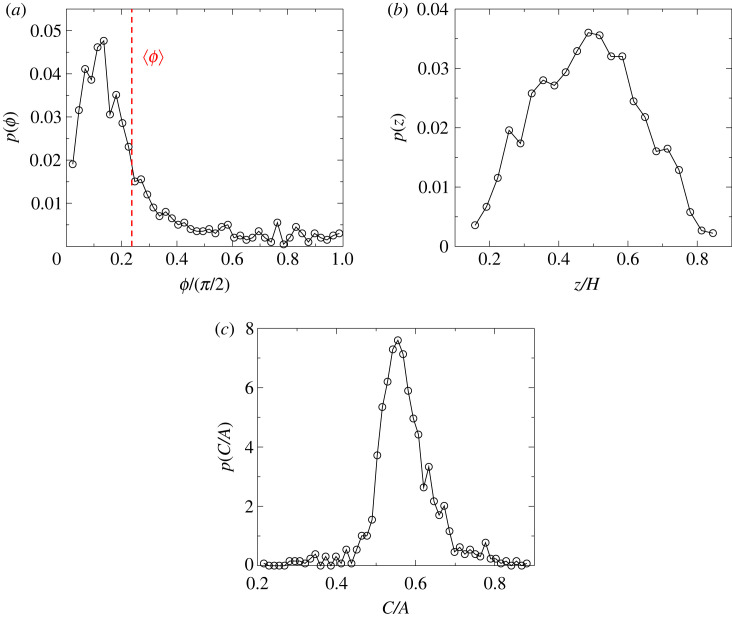
(*a*) Probability distribution *p*(*ϕ*) of the angle *ϕ* between the normal of the plane of the oblate ellipsoid, representing the fracture plane, and the load direction. The value of the average angle 〈*ϕ*〉 is highlighted by the vertical dashed line. (*b*) Probability distribution *p*(*z*) of the position *z* of the fracture plane along the vertical axis of the cylinder. (*c*) Probability distribution of the parameter *C*/*A* characterizing the sharpness of the fracture plane.

To obtain a deeper quantitative insight into the shape and orientation of the fracture plane, we determined the angle *ϕ* enclosed by the eigenvector of the moment of inertia matrix *I* corresponding to the smallest eigenvalue *C* and the direction of the external load (see figure 9*c* for illustration). With this definition *ϕ* is always positive, zero implies a normal parallel to the stress direction and *π*/2 perpendicular to it. The distribution *p*(*ϕ*) on figure 11*a* has its mode *ϕ*_*m*_ at the angle *ϕ*_*m*_/(*π*/2) ≈ 0.12 and the average value 〈*ϕ*/(*π*/2)〉 ≈ 0.22. The results imply that apart from some fluctuations the direction of the eigenvector is sub-parallel to the load direction. For a continuum, we would expect *ϕ* = 0, which is a local minimum in the probability distribution on figure 11*a* below the most likely sub-parallel orientation (mode) and the mean as shown. Thus all angles are possible, with a strong preference for sub-parallel orientations at low angles rather than the continuum solution *ϕ*/(2*π*) = 0. The sub-parallel orientation of the mean and mode indicate that material disorder (fluctuations) and the local dynamics (including interactions) have a discernible effect on the outcome. How sharply the fracture plane is defined can be characterized by the aspect ratio of the oblate ellipsoid, i.e. by the ratio of the smallest and largest eigenvalues *C*/*A* of the moment of inertia matrix *I*. The value of the aspect ratio *C*/*A* fluctuates in figure 11*c* but its distribution has a relatively sharp peak at *C*/*A* ≈ 0.55, which indicates that ellipsoid clouds of damage like the one presented in figure 9*c* are typical for the tensile failure of the uniaxially loaded cylindrical sample. Simulations showed that this behaviour emerges because the fracture path is composed of several planar segments which are somewhat shifted with respect to each other along the load direction. During the final catastrophic avalanche, these segments merge and form the spanning crack, which is not planar, again inconsistent with the continuum solution. The position *z* of the crack plane along the cylinder axis, approximated as the *z*-coordinate of the centre of mass of the catastrophic avalanche, equation (4.1), is randomly selected only during the fracture process, and cannot be predicted in advance from the micro-structure alone. This is illustrated in figure 11*b* where the distribution *p*(*z*) has a broad maximum centred at *z*/*H* ≈ 0.5.

Computer simulations in [31–33,37] showed that for uniaxial compression the spatial structure of damage undergoes a similar overall evolution, i.e. early stages of the fracture process are characterized by the random nucleation of small-sized micro-cracks scattered over the entire sample in an uncorrelated manner. As the system gradually approaches failure, spatial correlation of subsequent bursts emerges which leads to localization of damage. However, instead of a fracture plane, localization leads to the formation of an extended damage band [31–33,37]. Inside the band a large number of avalanches concentrate which leads to the complete fragmentation of the material with a power law distribution of fragment sizes [31,33]. Large-scale simulations in [33] revealed that the orientation of the damage band is determined by the internal friction coefficient of the material in agreement with experiments.

## 6. Discussion and conclusion

The fracture of heterogeneous materials is accompanied by the emission of crackling noise generated by avalanches of local breaking activity. AE measurements provide a deep insight into the dynamics of fracturing addressing even the possibility of forecasting the imminent catastrophic failure of loaded systems under certain conditions. Understanding the statistical features of cracking avalanches and the spatial structure of damage, furthermore, their dependence on the loading conditions, is essential both for the acoustic monitoring of engineering constructions and for field measurements on natural catastrophes such as earthquakes and landslides. Here, we performed a computational study of breaking avalanches generated during the fracture of porous rocks under tensile loading. Comparing the results to the corresponding outcomes of compression experiments obtained with the same specimens we wanted to isolate the effect of the loading condition on crackling noise in geomaterials.

Computer simulations revealed that the overall response of the specimen is significantly more brittle under tensile loading than under compression, because it has a higher ratio of yield stress to the ultimate strength and a faster drop down of the stress after failure in the tensile case. This means that the stress–strain curve has an overall linear character, and nonlinearity is only observed in the close vicinity of global failure. The critical strain where failure occurs proved to be only about one-third of the compressive strength of the same specimens. The fluctuating ultimate strength between starting materials obeys a Weibull distribution with a rather large value of the shape parameter confirming the mild fluctuations of strength values at the sample size considered. On the micro-scale fracture of the specimen proceeds in bursts which are driven by the gradual redistribution of load in the local neighbourhood of micro-cracks. Compared to the case of compressive failure, micro-cracking sets in earlier at a lower strain and the specimen can tolerate a lower amount of accumulated damage where a catastropic avalanche destroys the entire sample at a lower ultimate strength. Avalanches, identified as correlated trails of consecutive micro-cracking events, have a fluctuating size with a growing average as failure is approached. Our calculations revealed that under tensile loading a smaller number of avalanches occurs spanning a narrower range of size, duration and energy than during compression. This implies that under tensile loading the sample is more prone to system-scale failure in the sense that avalanches are more unstable leading to earlier and more sudden catastrophic collapse.

DEM simulations have the advantage that in addition to revealing the dynamics of fracturing they provide direct access to the spatial structure of damage. Our calculations showed that the beginning of the fracture process is dominated by the structural disorder giving rise to randomly dispersed small-sized avalanches all over the specimen. Spatial localization of avalanches is only observed in the close vicinity of macroscopic failure in such a way that a spatial region randomly emerges where the load concentration generated by breaking avalanches triggers further avalanches and eventually leads to the emergence of a catastrophic avalanche. As a consequence, a sharply defined fracture plane is formed along which the specimen breaks apart into two large pieces. Contrary to compressive failure [31,32,37] no damage band emerges where the material gets crushed. Instead, a localized damage zone formed of a cloud of tensile cracks emerges with an elliptical outline. The fracture plane in tension has a random position and orientation falling most likely in the centre of the cylindrical specimen and is oriented so that the normal to the best-fit ellipsoid is sub-parallel to the continuum solution of zero degrees. The continuum solution is a local minimum in the probability distribution of this angle for our simulations.

Single avalanches are characterized by their size, duration and energy dissipated during the avalanche. All quantities are found asymptotically to follow power law distributions with an exponential cut-off. Avalanches of larger size typically have a longer duration and a higher energy, which is described by a power law form of their correlation. Comparison of the avalanche exponents obtained under tension and compression revealed the robustness of the statistics of avalanche quantities. In spite of the substantially different micro-structure of damage and overall strength, there is no significant systematic difference of the statistics of breaking bursts between the two loading cases: the total number of avalanches and the range spanned by the avalanche size, duration and energy proved to be higher under compression but the power law exponents of tension and compression agree within the error bars. Our simulation results confirm the high degree of robustness of the statistical features of crackling noise with respect to the external loading conditions.

**Figure 12.**
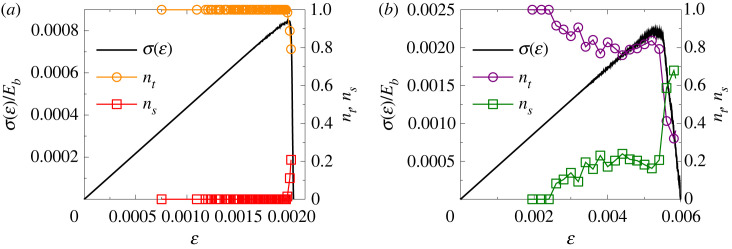
The fraction of beams failing dominantly under tension *n*_*t*_ and shear *n*_*s*_ are plotted as a function of strain ε for a single sample along with the corresponding constitutive curve σ(ε) for uniaxial tension (*a*) and compression (*b*). Note that $n_{t}(\varepsilon )+n_{s}(\varepsilon )=1$ holds for any ε.

By contrast, the loading configuration has a strong effect on the local failure mode of cohesive contacts inside the sample. The failure criterion equation (2.7) captures that the stretching and bending (shear) of a beam contribute to its breaking. The failure mode of a beam can be called tension or shear dominated if the first or second term of equation (2.7) is greater than the other one. The fraction of beams *n*_*t*_ and *n*_*s*_ of the tension and shear failure modes is plotted for a single sample in figure 12 as function of strain ε together with the constitutive curve for both loading cases. For uniaxial tensile loading (figure 12*a*) almost all the beams fail owing to local tension, and shear dominated failure only occurs in the close vicinity of final breakdown. Under uniaxial compression (figure 12*b*), the early uncorrelated cracking is dominated by tension, however, as fracture proceeds, shear-induced breaking more often occurs and it becomes dominating around the final localization in the damage band. The results are in agreement with experimental findings on fracture processes of porous rocks where the tensile and shear type of cracking could be discriminated [9]. Our results imply that these details of the local failure mode do not affect the overall statistics of avalanches, but they do play a decisive role in the temporal evolution of the burst sequence, which will be explored in a forthcoming publication.

## Data Availability

All data are accessible publicly at Dryad under the following link: https://doi.org/10.5061/dryad.nzs7h44wt [41].
